# The Association between Mental Health and Violence among a Nationally Representative Sample of College Students from the United States

**DOI:** 10.1371/journal.pone.0138914

**Published:** 2015-10-07

**Authors:** Joseph A. Schwartz, Kevin M. Beaver, J. C. Barnes

**Affiliations:** 1 School of Criminology and Criminal Justice, University of Nebraska at Omaha, Lincoln, Nebraska, United States of America; 2 College of Criminology and Criminal Justice, Florida State University, Tallahassee, Florida, United States of America; 3 Center for Social and Humanities Research, King Abdulaziz University, Jeddah, Saudi Arabia; 4 School of Criminal Justice, University of Cincinnati, Cincinnati, Ohio, United States of America; Shinshu University School of Medicine, JAPAN

## Abstract

**Objectives:**

Recent violent attacks on college campuses in the United States have sparked discussions regarding the prevalence of psychiatric disorders and the perpetration of violence among college students. While previous studies have examined the potential association between mental health problems and violent behavior, the overall pattern of findings flowing from this literature remain mixed and no previous studies have examined such associations among college students.

**Methods:**

The current study makes use of a nationally representative sample of 3,929 college students from the National Epidemiologic Study on Alcohol and Related Conditions (NESARC) to examine the prevalence of seven violent behaviors and 19 psychiatric disorder diagnoses tapping mood, anxiety, personality, and substance use disorders. Associations between individual and composite psychiatric disorder diagnoses and violent behaviors were also examined. Additional analyses were adjusted for the comorbidity of multiple psychiatric diagnoses.

**Results:**

The results revealed that college students were less likely to have engaged in violent behavior relative to the non-student sample, but a substantial portion of college students had engaged in violent behavior. Age- and sex-standardized prevalence rates indicated that more than 21% of college students reported at least one violent act. In addition, more than 36% of college students had at least one diagnosable psychiatric disorder. Finally, the prevalence of one or more psychiatric disorders significantly increased the odds of violent behavior within the college student sample.

**Conclusions:**

These findings indicate that violence and psychiatric disorders are prevalent on college campuses in the United States, though perhaps less so than in the general population. In addition, college students who have diagnosable psychiatric disorders are significantly more likely to engage in various forms of violent behavior.

## Introduction

In the early morning hours of November 20^th^, 2014, former Florida State University (FSU) student Myron May opened gunfire on the campus of FSU, paralyzing one student, and wounding two others. During this tragic event, May was involved in a shootout with campus and city police officers and eventually killed. In an unrelated event years earlier, Seung Hui Choi, a student at Virginia Tech University (VTU), gunned down 32 students and injured another 17 before committing suicide. In the wake of both the FSU and VTU shootings, reports began to emerge that the perpetrators had long and documented histories of mental illness; May being characterized by some as a paranoid schizophrenic and Choi being diagnosed with an anxiety disorder and major depression. As a direct result of the media coverage of these high-profile shootings—particularly the initial media reports about the mental health of the shooters—there has been growing concern regarding the intersection between mental health and violent behavior among college students [1–3].

Despite this general concern regarding the nexus between mental health and violence on university campuses, much of what is purportedly known about this association stems directly from non-empirical media reports. Empirical research examining mental health and violence among college students is virtually nonexistent. The limited research that has been published on these topics tends to be fragmented, focusing only on 1) mental health among college students [4–8] or 2) on the link between mental health and violence within non-university samples [9–13]. In perhaps the most rigorous assessment examining mental health among college students, Blanco et al. [5] analyzed data drawn from the National Epidemiologic Study on Alcohol and Related Conditions (NESARC). The results revealed that nearly 50% of all college-aged participants displayed symptoms of a diagnosable psychiatric disorder during the previous 12 months, a prevalence rate that was not statistically distinguishable from individuals who were similarly aged, but did not attend college. These findings directly converge with other studies examining the overall prevalence of mental health problems within the US college student population [6]. This study, however, did not provide any information about whether mental illness contributes to violence perpetrated by college students.

There is a large body of research that does examine the connection between mental health and violence in the general population. The results of these studies are far from unequivocal. Some findings reveal a significant association between psychiatric disorders in general and violence [14–17] whereas others fail to detect an association [12, 18–20]. More refined analyses, including the results of a comprehensive meta-analysis, document that specific psychiatric disorders increase the likelihood of certain violent behaviors for some people [10, 14, 15, 21–24]. This wide variation in findings can potentially be attributed to various study characteristics including the examination of non-representative samples (including institutionalized patients and incarcerated individuals) [12, 25], measurement ambiguity (including the use of self-reports documenting previously diagnosed disorders) [26], and inadequate controls for potentially confounding influences [27], among other limitations. In addition to these methodological limitations, no previous studies have directly examined whether any existing patterns of findings generalize to college students.

Given these gaps in the literature, the goal of the current study is to extend the existing literature by examining the potential association between mental health and violent behaviors among college students. In doing so, data are drawn from a nationally representative sample to examine differences in specific types of psychiatric disorders and violent behaviors between college students and their non-student counterparts. Additional analyses examine whether diagnoses of specific psychiatric disorders are associated with specific types of violent behaviors among college students. To our knowledge, this is the first study to examine such associations within a nationally representative sample of college students.

## Materials and Methods

### Sample

The current study analyzes a sample of self-identified college students from Wave 1 of the NESARC, a longitudinal and nationally representative sample of adults aged 18 years or older in the United States. Data collection was carried out by trained representatives of the U.S. Census Bureau and supervised by the National Institute on Alcohol Abuse and Alcoholism (NIAAA). All NESARC respondents provided written informed consent and the research protocol of the NESARC study was approved by the U.S. Census Bureau and U.S. Office of Management and Budget. Additional information regarding the employed sampling procedures has been provided in detail in previous reports [28]. The first wave of interviews was collected using a computer-based survey instrument between 2001 and 2002 and included a total of 43,093 respondents [28]. The NESARC achieved a household response rate of 89%, an individual response rate of 93% and an overall response rate of 81% at Wave 1. A second round of interviews was completed approximately four years later, between 2004 and 2005, and included 34,653 respondents from the initial sample.

Nested within the NESARC sample is a nationally representative sample of college students. During wave 1 interviews, respondents were asked a series of questions to determine their current student status. First, respondents were asked whether they were a full-time or part-time student in the past 12 months. Respondents who affirmed their student status were then asked to report their grade level during the 2000–2001 school year. Based on responses to this question, the subsample of respondents who were enrolled in either undergraduate or graduate classes was identified. A total of 6,236 students were nested in the full sample, with 1,155 (18.5% of all students) students still in high school or a graduate equivalency degree (GED) program, 3,097 (49.7%) students enrolled in an undergraduate program, and 832 (13.3%) students enrolled in a graduate program. In addition, 1,152 (18.5%) students identified their grade level during the 2000–2001 school year as “other,” indicating that they were not enrolled in any of the provided categories. The student subsample analyzed in the current study was restricted to respondents who identified themselves as undergraduate or graduate college students (*n* = 3,929 students) and had complete data on all study measures. Due to the amount of time that lapsed between the Wave 1 and Wave 2 interviews, many individuals who were enrolled in college during the first wave of interviews were no longer enrolled during the second wave. Based on this reduction in sample size coupled with the relatively low prevalence of the examined mental health problems, only measures collected during Wave 1 interviews were included in the current study. Key demographic measures for both the college student and non- student subsamples of the NESARC, along with information on the included study covariates, are summarized in Table 1.

**Table 1:** Sample Characteristics Reported by College Status.

	Prevalence		Odds Ratios		
	*Not in College*	*College Student*			
	*90*.*07%*	*9*.*93%*			
	*(N = 39*,*164)*	*(N = 3*,*929)*	*OR*	*95% CI*	*p*
**Mental Disability**					
Bottom 25th Percentile, % (n)	23.53 (9,812)	22.83 (880)	.96	.87; 1.06	.438
26th to 49th Percentile, % (n)	25.14 (9,508)	30.71 (1,180)	**1.32**	**1.21; 1.44**	**< .001**
50th to 74th Percentile, % (n)	26.53 (9,761)	27.34 (1,051)	1.04	.94; 1.16	.446
Top 25th Percentile, % (n)	24.79 (9,764)	19.13 (787)	**.72**	**.65; .80**	**< .001**
**General Health**					
Bottom 25th Percentile, % (n)	16.09 (7,208)	5.00 (235)	**.27**	**.23; .33**	**< .001**
26th to 49th Percentile, % (n)	24.72 (9,969)	15.85 (684)	**.57**	**.51; .64**	**< .001**
50th to 74th Percentile, % (n)	29.64 (11,094)	35.18 (1,335)	**1.29**	**1.18; 1.41**	**< .001**
Top 25th Percentile, % (n)	29.56 (10,893)	43.97 (1,675)	**1.87**	**1.70; 2.06**	**< .001**
**Stressful Life Experiences**					
Bottom 25th Percentile, % (n)	31.54 (12,389)	22.74 (877)	**.64**	**.58; .70**	**< .001**
26th to 49th Percentile, % (n)	24.95 (9,478)	21.18 (824)	**.81**	**.73; .90**	**< .001**
50th to 74th Percentile, % (n)	21.10 (8,117)	19.64 (767)	.91	.83; 1.01	.078
Top 25th Percentile, % (n)	22.41 (8,594)	36.45 (1,409)	**1.99**	**1.82; 2.16**	**< .001**
**Family Income, mean (SD)**	10.35 (4.88)	9.24 (5.07)	—	—	—
**Living Situation**					
Living with Family % (n)	—	24.91 (912)	—	—	—
Living away from Family, % (n)	—	75.09 (3,017)	—	—	—
**Marital Status**					
Married, % (n)	60.93 (19,580)	33.14 (1,189)	**.32**	**.29; .35**	**< .001**
Not Married, % (n)	39.07 (19,584)	66.86 (2,740)	**3.15**	**2.84; 3.48**	**< .001**
**Sex**					
Male, % (n)	48.11 (16,945)	46.15 (1,573)	.92	.85; 1.01	.066
Female, % (n)	51.89 (22,219)	53.85 (2,356)	1.08	.99; 1.18	.066
**Race**					
White, % (n)	71.35 (22,469)	66.61 (2,038)	**.80**	**.73; .88**	**< .001**
Black, % (n)	10.77 (7,382)	13.87 (863)	**1.33**	**1.19; 1.50**	**< .001**
Native American, % (n)	2.18 (649)	1.61 (52)	.73	.53; 1.02	.067
Asian, % (n)	4.05 (1,124)	7.26 (208)	**1.85**	**1.51; 2.82**	**< .001**
Hispanic, % (n)	11.66 (7,540)	10.65 (768)	.90	.78; 1.04	.161
**Age**					
18 to 25 years old, % (n)	10.73 (3,963)	52.95 (1,875)	**9.36**	**8.44; 10.38**	**< .001**
26 to 33 years old, % (n)	14.16 (5,418)	20.12 (859)	**1.53**	**1.37; 1.70**	**< .001**
34 to 41 years old, % (n)	17.20 (6,785)	12.53 (581)	**.69**	**.61; .78**	**< .001**
42 years or older, % (n)	57.91 (22,998)	14.40 (614)	**.12**	**.11; .14**	**< .001**

Abbreviations: OR, odds ratio; CI, confidence interval; SD, standard deviation

All estimates calculated using survey weights to correct for sampling procedures. Bolded coefficients have an accompanying 95% CI that does not include 1.00.

### Measures

#### Violent behavior measures

During Wave 1 interviews, respondents were asked to report their involvement in 33 different antisocial behaviors. For each behavior, respondents were asked to indicate whether they had ever engaged in the behavior with responses coded dichotomously (0 = *no*, 1 = *yes*). The results of a confirmatory factor analysis (CFA) model revealed that seven of the 33 items loaded on a single violent behavior factor for both the full NESARC sample (comparative fit index [CFI] = .987, Tucker-Lewis Index [TLI] = .981, root mean square error of approximation [RMSEA] = .017) and the college student subsample (CFI = .990, TLI = .986, RMSEA = .016). These seven measures possessed strong face validity and included the following behaviors: bullying others; frequently getting into and instigating fights; physical fighting with a significant other; using a weapon in a fight; hitting someone so hard they needed medical attention; harassing, threatening, or blackmailing someone; and physically hurting another person on purpose. In addition to the seven violent behavior measures identified in the CFA model, a dichotomous violent behavior measure that identified individuals who engaged in *any* of the seven violent behaviors was also included in the analysis. All violence measures included in the current study are listed and summarized in Table 2.

**Table 2:** Standardized Prevalence for Violence Psychiatric Disorder Measures by College Status.

	Standardized Prevalence		Adjusted Odds Ratio		
	*Not in College*	*College Student*			
	*90*.*07%*	*9*.*93%*			
	*(N = 39*,*164)*	*(N = 3*,*929)*	*AOR*	*95% CI*	*p*
**Violence Measures**					
Bullied Others, % (n)	9.64 (2,180)	8.00 (280)	**.71**	**.58; .88**	**.002**
Got into Fights, % (n)	4.55 (1,022)	2.80 (104)	**.55**	**.42; .72**	**< .001**
Domestic Violence, % (n)	8.23 (2,795)	6.78 (262)	**.65**	**.55; .77**	**< .001**
Used Weapon in Fight, % (n)	3.63 (1,078)	3.67 (117)	**.75**	**.58; .99**	**.040**
Hit Someone Injury, % (n)	10.45 (2,070)	9.75 (258)	**.81**	**.66; .98**	**.032**
Harass/Threatened Someone, % (n)	3.20 (551)	3.43 (120)	.98	.73; 1.32	.915
Physically Injured Someone, % (n)	8.60 (1,685)	9.14 (274)	.94	.77; 1.16	.564
Any Violent Behavior, % (n)	22.53 (6,222)	21.35 (766)	**.82**	**.71; .94**	**.006**
**Mood Disorder Diagnosis**					
Major Depression, % (n)	9.10 (2,739)	8.36 (380)	.96	.84; 1.09	.516
Dysthymia, % (n)	2.03 (781)	1.81 (62)	.84	.59; 1.20	.340
Manic Disorder, % (n)	2.54 (637)	2.01 (87)	.79	.58; 1.09	.152
Hypomanic Disorder, % (n)	2.56 (388)	2.27 (92)	.92	.67; 1.27	.618
Any Mood Disorder, % (n)	11.90 (3,560)	10.95 (505)	.96	.84; 1.09	.484
**Anxiety Disorder Diagnosis**					
Panic Disorder, % (n)	2.04 (584)	1.87 (69)	.96	.69; 1.32	.777
Panic Disorder w/ Agro., % (n)	0.75 (232)	1.14 (22)	.96	.55; 1.69	.896
Social Phobia, % (n)	3.07 (1,021)	3.09 (119)	1.04	.77; 1.40	.808
Specific Phobia, % (n)	8.35 (2,748)	8.19 (325)	.97	.83; 1.12	.643
Generalized Anxiety, % (n)	3.68 (1,378)	4.25 (126)	1.04	.81; 1.35	.719
Any Anxiety Disorder, % (n)	13.38 (4,689)	13.32 (519)	.99	.87; 1.13	.922
**Personality Disorder Diagnosis**					
Conduct Disorder, % (n)	1.67 (366)	2.11 (49)	.89	.53; 1.49	.658
Antisocial Personality, % (n)	6.28 (1,249)	5.92 (173)	.82	.64; 1.05	.116
Avoidant Personality, % (n)	3.15 (905)	2.50 (90)	**.71**	**.53; .95**	**.022**
Dependent, % (n)	0.76 (193)	0.34 (15)	**.48**	**.26; .90**	**.022**
Obsessive-Compulsive, % (n)	8.21 (2,891)	9.93 (370)	1.17	.99; 1.37	.061
Paranoid Personality, % (n)	6.31 (1,884)	4.90 (221)	**.71**	**.57; .88**	**.002**
Schizoid Personality, % (n)	3.88 (1,277)	3.13 (148)	.78	.60; 1.01	.062
Histrionic Personality, % (n)	3.05 (695)	2.71 (113)	.88	.68; 1.14	.340
Any Personality Disorder, % (n)	18.28 (5,863)	19.15 (743)	.97	.86; 1.10	.685
**Substance Use Disorder Diagnosis**					
Alcohol Use, % (n)	14.50 (2,807)	16.22 (520)	**1.17**	**1.02; 1.35**	**.027**
Drug Use, % (n)	5.48 (645)	3.94 (122)	**.73**	**.57; .95**	**.018**
Any Substance Use Disorder, % (n)	16.22 (3,111)	17.34 (567)	1.11	.97; 1.27	.114
**Any Diagnosis, % (n)**	35.55 (11,814)	36.77 (1,525)	1.06	.97; 1.15	.195

Abbreviations: AOR, adjusted odds ratio; CI, confidence interval; Agro, agroaphobia

All estimates calculated using survey weights to correct for sampling procedures. Age and sex standardized prevalence presented. Bolded coefficients have an accompanying 95% CI that does not include 1.00.

#### Psychiatric disorder measures

The NESARC also contains the Alcohol Use Disorder and Associated Disability Interview Schedule—DSM-IV Version (AUDADIS-IV), which is a self-report instrument aimed at diagnosing a large number of psychiatric disorders based on criteria specified in the *Diagnostic and Statistical Manual of Mental Disorders*, *Fourth Edition* [29]. The inclusion of the AUDADIS-IV allowed for the creation of a wide range of diagnostic measures tapping mental health and substance use problems, along with personality disorders, during the year prior to Wave 1 interviews. Four mood disorder diagnoses were assessed: major depressive disorder; dysthymia; manic disorder; and hypomanic disorder. In addition, five anxiety disorders were included: panic disorder (with and without agoraphobia); social phobia; specific phobia; and generalized anxiety disorder. Importantly, all mood and anxiety diagnosis measures were adjusted to rule out the contributing factors of illness and substance use by the NESARC team [26]. This procedure was carried out by ensuring that respondents were not physically ill and abstained from drug and alcohol use during the time period in which the diagnosis was made. Eight personality disorders were also assessed: conduct disorder; antisocial personality disorder; avoidant disorder; dependent disorder; obsessive-compulsive disorder; paranoid personality disorder; schizoid personality disorder; and histrionic personality disorder. Finally, two substance use disorder diagnoses tapping alcohol and drug use disorders were also included. In addition to each of the individual diagnosis measures, five composite diagnosis indicators were also included in the analysis: any mood disorder; any anxiety disorder; any personality disorder; any substance use disorder; and any psychiatric disorder. Additional information on the creation of any of the mental health measures included in the NESARC can be found in previous studies [28, 30]. A complete list of psychiatric disorder measures included in the current study can be found in Table 2.

#### Statistical confounders

In an effort to isolate the effect of the mental health diagnosis on violent behavior, nine potential confounder variables were included in the statistical models. The mental disability and general health subsets of the Short Form 12 Health Survey Version 2 (SF-12v2), both of which were norm-based according to age and sex, were included in the analysis. A stressful life events index was created using responses to 12 items asking about the prevalence of various stressful events within the past year including someone close to you dying, experiencing a major financial crisis, or being the victim of a crime. All 12 items were summed and then coded into quartiles with higher scores indicating a greater level of stressful events in the past year. A measure of total family income was coded across 21 categories, with greater values indicating greater overall family income (1 = *less than $5*,*000 per year*; 21 = *greater than $200*,*000 per year*). A dichotomous measure identifying students who lived away from family while attending school was also included. In addition, a dichotomous measure of marital status (0 = *not married*; 1 = *married*) was included. Finally, three demographic covariates were also included in the analysis. Sex was coded so that 0 = *female* and 1 = *male*; race was coded as a series of dummy variables with Caucasian as the reference category. Due to the employed standardization procedures (discussed in more detail below), age was measured categorically in eight year increments and coded such that 1 = *18–25 years old*; 2 = *26–33 years old*; 3 = *34–41 years old*; and 4 = *42 years old or older*.

### Statistical Analysis

All analyses were performed in the statistical software program, *Stata* (version 13.1) using properly specified sample weights, which adjusted standard errors and confidence intervals to account for the complex design features of the NESARC [28]. The first step of the analysis estimated the prevalence of violent behavior and psychiatric disorders for both the college student subsample and all respondents who were not enrolled in college during Wave 1 interviews. Simply comparing unstandardized prevalence rates between two groups that possess significant demographic differences would result in biased estimates. As would be expected, the average age of the non-student sample (mean = 46.97, median = 45, range = 18–98) was significantly greater (*F* = 3335.35, *p* < .001) than the average age of the college student sample (mean = 29.10, median = 25, range = 18–86). In addition, sex differences between the student and non-student subsamples were only marginally-significant (*F* = 3.50, *p* = .066), but based on the substantial number of previous studies reporting significant sex-differences in both violent behavior and psychiatric disorders [10] such differences also warrant attention. Based on these observations, the prevalence rates reported for the violent behavior measures and the psychiatric disorder measures were standardized by age and sex. In an effort to better display the overall impact of the standardization procedures, the unstandardized and standardized prevalence rates for the violent behavior and psychiatric disorder measures are presented in S1 Table.

Second, two sets of binary logistic regression equations were used to estimate the association between each psychiatric disorder diagnosis and violent behavior. The first set of estimated models examined the association between each included psychiatric disorder measure and the violent behavior outcomes. This same set of models was estimated again but was adjusted for the possible comorbidity of multiple disorders by including all other composite psychiatric diagnosis measures in the equation. For example, when estimating the association between major depressive disorder (a mood disorder) and bullying, the anxiety, personality, and substance use disorder composite measures were included in the equation. The second set of logistic regression models focused on the association between the composite psychiatric disorder measures and the violent behavior outcomes. Once again, models that were both unadjusted and adjusted for comorbidity were estimated. The third step of the analysis involved visually displaying the results of the logistic regression equations estimating the association between the composite psychiatric disorder measure and the violent behavior measures.

The fourth and final step of the analysis involved estimating an additional set of logistic regression models aimed at comparing the potential association between the composite psychiatric disorder measures and the composite violent behavior measure across student status. This step was employed to assess whether any detected associations significantly varied between the student and non-student subsamples. The estimated models included the examined composite psychiatric disorder measure and a dichotomous student indicator, along with a multiplicative interaction term between both measures. A significant interaction term would provide preliminary evidence that any detected association significantly differed across the two subsamples. Importantly, statistical confounders were included in all of the estimated logistic regression equations.

## Results

Table 2 displays the prevalence (standardized by age and sex) of the violent behavior and psychiatric disorder measures for the college and non-college subsamples along with the accompanying cell sizes. Importantly, due to the use of sample weights and standardization procedures, the reported cell sizes do not directly correspond to the reported prevalence rates. To determine whether observed discrepancies significantly varied between the two subsamples, age- and sex-adjusted odds ratios were estimated by separately regressing the violence and psychiatric disorder measures on a dichotomous college student indicator (0 = *non-student*, 1 = *student*), age, and sex. The adjusted odds ratios, along with their accompanying 95% confidence intervals and *p*-values, are presented in Table 2. Hitting someone so hard they needed medical attention (9.75%) and physically injuring someone on purpose (9.14%) were the most common forms of violent behavior among college students, while hitting someone so hard they needed medical attention (10.45%) and bullying others (9.64%) were the most common forms of violence in the non-student sample. In addition, a sizable proportion of both populations engaged in at least one form of violent behavior, with 21.35% of the college student sample and 22.53% of their non-student counterparts reporting at least one violent incident. The results of the logistic regression models comparing the prevalence of both subsamples revealed that the non-student sample consistently engaged in more violent behavior relative to the college student sample with two exceptions, engaging in threatening/harassing behaviors (AOR = .98; 95% CI = .73–1.32) and physically injured someone (AOR = .94; 95% CI = .77–1.16) did not significantly differ between the two groups. However, when examining the overall prevalence of violent behavior, non-students engaged in significantly greater overall levels (AOR = .82; 95% CI = .71-.94).

Table 2 also displays the standardized prevalence of the psychiatric disorder diagnoses for the student and non-student subsamples, along with the age- and sex-adjusted odds ratios comparing the prevalence between the two subsamples. Within both samples, major depression was the most common mood disorder (non-student sample = 9.10%; student sample = 8.36%), the presence of a specific phobia was the most common anxiety disorder (non-student sample = 8.35%; student sample = 8.19%), obsessive-compulsive disorder was the most common personality disorder (non-student sample = 8.21%; student sample = 9.93%), and alcohol use disorder was the most common substance use disorder (non-student sample = 14.50%; student sample = 16.22%). In addition, 35.55% of the non-student sample and 36.77% of the student sample suffered from at least one psychiatric disorder. The results of the sample comparisons revealed that the majority of the examined disorders did not significantly differ between the non-student and student samples. The only discrepancy from this overall pattern of findings were present among the personality and substance use disorder measures in which avoidant personality (AOR = .71; 95% CI = .53-.95), dependent personality (AOR = .48; 95% CI = .26-.90), paranoid personality (AOR = .71; 95% CI = .57-.88), and drug use (AOR = .73; 95% CI = .57-.95) disorders were all more prevalent within the non-student sample, while alcohol use disorder was more prevalent in the college student sample (AOR = 1.17; 95% CI = 1.02–1.35). Despite such differences, the composite mood, anxiety, personality, substance, and psychiatric disorder measures were not significantly different between the two samples.

The next step of the analysis was to estimate the association between the individual psychiatric disorder diagnoses and violent behavior within the college student subsample. Recall that logistic regression models were estimated because the violent behavior outcome variables were all coded dichotomously. The resulting odds ratios, adjusted for each of the included statistical confounders, are presented in Table 3. Due to the large number of estimated models (19 psychiatric disorder measures * 8 violent behavior outcomes = 152 comparisons), the alpha-level was adjusted using a Bonferroni correction (.05/152 = .00033). A total of eight psychiatric disorder measures were significantly associated with engaging in *any* violent behavior (see the last column of Table 3): major depression (OR = 1.99; 95% CI = 1.42–2.79); manic disorder (OR = 2.91; 95% CI = 1.68–5.03); conduct disorder (OR = 9.50; 95% CI = 3.18–28.38); antisocial personality disorder (OR = 11.18; 95% CI = 5.76–21.68); obsessive-compulsive personality disorder (OR = 2.00; 95% CI = 1.44–2.76); paranoid personality disorder (OR = 2.79; 95% CI = 1.71–4.55); histrionic personality disorder (OR = 4.50; 95% CI = 2.67–7.58); and alcohol use disorder (OR = 2.13; 95% CI = 1.64–2.77). Directly in line with this pattern of results, these same psychiatric diagnoses were significantly associated with individual violent behaviors. Of note is that the coefficient of relationship was *positive* across all of the associations, indicating that the presence of a psychiatric disorder *increases* the odds of violent behavior.

**Table 3:** Adjusted Odds Ratios for Psychiatric Diagnoses and Violent Behavior.

	AOR for Violent Behavior (95% CI)							
	Bullied	Fighting	Domestic Violence	Weapon	Hit Hard	Harass/ Threaten	Injured Someone	Any Violence
**Mood Disorders**								
Major Depression	**1.86**	1.70	**1.81**	1.08	**2.47**	**1.85**	**1.81**	**1.99** ^a^
	**1.21; 2.86**	.98; 2.96	**1.14; 2.88**	.57; 2.03	**1.49; 4.09**	**1.04; 3.31**	**1.11; 2.95**	**1.42; 2.79**
Dysthymia	**2.59**	2.68	1.03	**3.31**	1.11	**8.55** ^a^	1.74	1.61
	**1.02; 6.59**	.90; 7.94	.39; 2.73	**1.10; 9.98**	.38; 3.19	**3.47; 21.08**	.61; 5.00	.80; 3.28
Manic	**2.66**	**6.05** ^a^	**2.77**	1.09	**2.77**	**2.34**	1.23	**2.91** ^a^
	**1.56; 4.51**	**2.85; 12.84**	**1.39; 5.52**	.37; 3.19	**1.23; 6.26**	**1.11; 4.92**	.55; 2.79	**1.68; 5.03**
Hypomanic	1.00	2.56	1.51	**3.22**	1.45	2.58	1.61	**1.85**
	.45; 2.20	1.00; 6.55	.62; 3.66	**1.39; 7.44**	.64; 3.32	.99; 6.68	.85; 3.05	**1.12; 3.04**
**Anxiety Disorders**								
Panic	2.23	1.51	1.32	1.60	1.37	1.85	.84	1.51
	.93; 5.35	.38; 6.04	.46; 3.75	.39; 6.60	57; 3.34	.35; 9.80	.29; 2.45	.77; 2.99
Panic w/ Agoraphobia	1.42	—^b^	1.82	.99	2.99	.27	.30	1.45
	.39; 5.11		.58; 5.69	.13; 7.39	.85; 10.48	.03; 2.22	.04; 2.38	.54; 3.92
Social Phobia	.89	**.21**	1.56	.49	1.52	.46	1.67	1.42
	.38; 2.08	**.04; 1.00**	.73; 3.30	.13; 1.78	.61; 3.81	.12; 1.68	.74; 3.76	.82; 2.44
Specific Phobia	**1.72**	1.87	**1.75**	**2.85**	1.44	**2.21**	1.34	**1.76**
	**1.07; 2.76**	.97; 3.61	**1.06; 2.88**	**1.40; 5.78**	.86; 2.42	**1.18; 4.13**	.81; 2.21	**1.22; 2.53**
Generalized Anxiety	**2.15**	2.31	1.57	1.11	1.73	2.40	**2.40**	**1.71**
	**1.12; 4.13**	.92; 5.79	.84; 2.93	.43; 2.89	.83; 3.60	.94; 6.10	**1.23; 4.67**	**1.03; 2.86**
**Personality Disorders**								
Conduct Disorder	**5.34**	**4.34**	.64	1.69	.85	**3.93**	**9.31** ^a^	**9.50** ^a^
	**2.08; 13.69**	**1.74; 10.85**	.16; 2.56	.51; 5.55	.24; 3.07	**1.06; 14.54**	**4.23; 20.50**	**3.18; 28.38**
Antisocial	**11.20** ^a^	**18.04** ^a^	**5.02** ^a^	**11.33** ^a^	**6.62** ^a^	**12.63** ^a^	**5.20** ^a^	**11.18** ^a^
	**7.33; 17.12**	**10.38; 31.34**	**2.84; 8.90**	**6.23; 20.58**	**3.78; 11.58**	**7.21; 22.11**	**3.18; 8.50**	**5.76; 21.68**
Avoidant	1.89	.58	1.14	.40	1.16	1.20	1.08	1.52
	.82; 4.34	.23; 1.50	.59; 3.38	.10; 1.61	.39; 3.47	.36; 4.00	.40; 2.92	.83; 2.79
Dependent	3.18	2.23	2.30	—^b^	2.82	4.57	.84	1.93
	.65; 15.58	.18; 27.90	.66; 8.00		.73; 10.88	.76; 27.50	.10; 6.90	.68; 5.48
Obsessive-Compulsive	1.41	**2.52**	1.46	**2.63**	**2.28**	**2.94** ^a^	**1.73**	**2.00** ^a^
	.92; 2.17	**1.35; 4.67**	.91; 2.35	**1.44; 4.78**	**1.39; 3.74**	**1.78; 4.86**	**1.11; 2.68**	**1.44; 2.76**
Paranoid	**2.90** ^a^	**2.82**	**2.00**	**2.03**	**2.13**	**3.39**	**2.08**	**2.79** ^a^
	**1.69; 4.98**	**1.41; 5.69**	**1.24; 3.24**	**1.01; 4.04**	**1.15; 3.98**	**1.66; 6.93**	**1.14; 3.80**	**1.71; 4.55**
Schizoid	1.33	**2.62**	.99	2.11	**2.31**	**2.27**	1.05	1.46
	.72; 2.45	**1.20; 5.78**	.50; 1.93	87; 5.14	**1.20; 4.45**	**1.03; 5.02**	.55; 2.00	.95; 2.24
Histrionic	**3.92** ^a^	**2.85**	**4.97** ^a^	**3.00**	**2.24**	**4.33** ^a^	**4.73** ^a^	**4.50** ^a^
	**2.10; 7.33**	**1.27; 6.39**	**2.85; 8.68**	**1.34; 6.73**	**1.18; 4.27**	**2.07; 9.08**	**2.50; 8.95**	**2.67; 7.58**
**Substance Use Disorders**								
Alcohol Use	**1.80**	1.83	**1.61**	**2.74**	**2.18**	1.40	**1.97**	**2.13** ^a^
	**1.17; 2.75**	.99; 3.37	**1.04; 2.49**	**1.49; 5.03**	**1.40; 3.39**	.77; 2.56	**1.33; 2.91**	**1.64; 2.77**
Drug Use	**2.16**	**2.89**	**2.67**	1.28	1.43	1.51	**2.26**	**2.38**
	**1.17; 4.00**	**1.38; 6.05**	**1.38; 5.18**	.48; 3.41	.69; 2.94	.59; 3.89	**1.25; 4.07**	**1.37; 4.13**

All odds ratios estimated using sampling weights and were adjusted for the following confounders: mental disability; general health; stressful life events; family income; living situation; marital status; sex; race; and age. Bolded coefficients have an accompanying 95% CI that does not include 1.00.

^a^ *p <* .00033 (Bonferroni corrected alpha for 152 comparisons)

^b^Odds ratios could not be estimated due to insufficient cell size

The results of models adjusted for comorbidity (and all other statistical confounders) are presented in Table 4. The overall pattern of results was similar to the unadjusted models aside from the number of significant associations detected. In total, four psychiatric disorder measures remained significantly associated with engaging in any violent behavior after controlling for comorbidity: conduct disorder (OR = 9.77; 95% CI = 3.24–29.52); antisocial personality disorder (OR = 9.97; 95% CI = 5.00–19.85); histrionic personality disorder (OR = 3.46; 95% CI = 2.02–5.92); and alcohol use disorder (OR = 2.04; 95% CI = 1.55–2.67).

**Table 4:** Adjusted Odds Ratios for Psychiatric Diagnoses and Violent Behavior with Controls for Comorbidity.

	AOR for Violent Behavior (95% CI)^a^							
	Bullied	Fighting	Domestic Violence	Weapon	Hit Hard	Harass/ Threaten	Injured Someone	Any Violence
**Mood Disorders**								
Major Depression	1.26	1.08	1.41	.64	**1.92**	1.21	1.33	1.46
	.78; 2.01	.59; 1.98	.86; 2.30	.32; 1.30	**1.11; 3.33**	.64; 2.28	.78; 2.25	.99; 2.14
Dysthymia	1.38	1.25	.72	1.99	.68	**4.60**	1.00	.92
	.55; 3.44	.40; 3.88	.27; 1.92	.59; 6.71	.21; 2.24	**1.87; 11.34**	.34; 3.02	.43; 1.97
Manic	1.47	**3.50**	**2.00**	.56	1.71	1.40	.69	1.74
	.83; 3.61	**1.43; 8.60**	**1.02; 3.92**	.17; 1.86	.74; 3.98	.57; 3.44	.30; 1.60	.92; 3.31
Hypomanic	.69	1.76	1.10	2.22	1.09	1.86	1.21	1.37
	.32; 1.51	.61; 5.06	.46; 2.64	.90; 5.46	.46; 2.55	.64; 5.42	.64; 2.30	.83; 2.25
**Anxiety Disorders**								
Panic	1.52	.86	.89	1.07	.84	1.04	.53	.96
	.57; 4.05	.19; 3.89	.33; 2.37	.20; 5.58	.30; 2.34	.18; 5.89	.15; 1.84	.51; 1.80
Panic w/ Agoraphobia	.86	—^b^	1.30	.68	1.92	.13	.19	.85
	.23; 3.22		.45; 3.78	.09; 5.04	.55; 6.65	.02; 1.14	.02; 1.67	.32; 2.26
Social Phobia	.42	**.08**	.93	.27	.86	**.18**	1.00	.70
	.17; 1.00	**.02; .41**	.40; 2.14	.07; 1.09	.31; 2.35	**.05; .65**	.44; 2.26	.38; 1.28
Specific Phobia	1.12	1.14	1.38	**2.21**	1.07	1.41	.95	1.23
	.67; 1.90	.59; 2.20	.84; 2.27	**1.07; 4.56**	.61; 1.86	.76; 2.61	.55; 1.63	.82; 1.84
Generalized Anxiety	1.37	1.26	1.11	.77	1.12	1.25	1.65	1.09
	.71; 2.64	.46; 3.45	.56; 2.18	.28; 2.09	.52; 2.43	.42; 3.72	.79; 3.42	.61; 1.93
**Personality Disorders**								
Conduct Disorder	**5.37**	**4.16**	.60	1.70	.83	**3.74**	**9.50** ^a^	**9.77** ^a^
	**2.12; 13.53**	**1.67; 10.32**	.15; 2.43	.52; 5.59	.25; 2.82	**1.12; 12.47**	**4.22; 21.41**	**3.24; 29.52**
Antisocial	**10.17** ^a^	**16.76** ^a^	**4.52** ^a^	**10.40** ^a^	**5.90** ^a^	**11.68** ^a^	**4.61** ^a^	**9.97** ^a^
	**6.56; 15.76**	**9.42; 29.78**	**2.58; 7.89**	**5.69; 19.00**	**3.25; 10.72**	**6.80; 20.06**	**2.71; 7.82**	**5.00; 19.85**
Avoidant	1.52	.41	1.17	.33	.90	.80	.86	1.16
	.69; 3.62	.14; 1.18	.49; 2.79	.07; 1.52	.27; 3.03	.21; 2.99	.31; 2.36	.60; 2.24
Dependent	2.26	1.66	1.63	—^b^	2.04	3.03	.48	1.07
	.39; 13.06	.16; 16.92	.51; 5.19		.61; 6.79	.37; 24.68	.06; 4.11	.37; 3.12
Obsessive-Compulsive	1.25	**2.20**	1.31	**2.47**	**2.10**	**2.53**	1.59	**1.82**
	.77; 2.10	**1.13; 4.26**	.81; 2.12	**1.34; 4.57**	**1.25; 3.51**	**1.52; 4.23**	.99; 2.54	**1.29; 2.56**
Paranoid	**2.38**	2.10	1.61	1.65	1.64	**2.58**	1.65	**2.13**
	**1.39; 4.09**	1.00; 4.42	.99; 2.61	.80; 3.41	.85; 3.17	**1.22; 5.46**	.89; 3.07	**1.27; 3.57**
Schizoid	1.07	2.02	.76	1.86	1.99	1.67	.87	1.16
	.54; 2.12	.81; 5.09	.37; 1.55	.72; 4.79	1.00; 3.98	.72; 3.84	.41; 1.84	.74; 1.81
Histrionic	**3.19**	2.22	**4.10** ^a^	2.34	1.74	**3.52**	**3.80** ^a^	**3.46** ^a^
	**1.68; 6.08**	.90; 5.45	**2.34; 7.17**	.95; 5.79	.88; 3.42	**1.65; 7.52**	**1.96; 7.33**	**2.02; 5.92**
**Substance Use Disorders**								
Alcohol Use	**1.60**	1.74	**1.56**	**2.58**	**2.02**	1.28	**1.88**	**2.04** ^a^
	**1.05; 2.45**	.94; 3.19	**1.00; 2.42**	**1.40; 4.78**	**1.28; 3.17**	.71; 2.30	**1.24; 2.85**	**1.55; 2.67**
Drug Use	**1.89**	**2.45**	**2.42**	1.15	1.26	1.17	**2.08**	2.21
	**1.01; 3.53**	**1.08; 5.56**	**1.20; 4.87**	.40; 3.31	.58; 2.77	.41; 3.28	**1.07; 4.03**	1.22; 4.03

All odds ratios estimated using sampling weights and were adjusted for the following confounders: mental disability; general health; stressful life events; family income; living situation; marital status; sex; race; and age. Odds ratios also adjusted for all other composite psychiatric diagnoses. Bolded coefficients have an accompanying 95% CI that does not include 1.00.

^a^ *p <* .00033 (Bonferroni corrected alpha for 152 comparisons)

^b^Odds ratios could not be estimated due to insufficient cell size

The next step in the analysis examined the association between the composite psychiatric disorder measures and the violent behavior outcomes both before and after adjusting for comorbidity. The results of all estimated models are presented in Table 5. In an effort to better contextualize the magnitude of the association between the composite mental health disorders and the violence outcomes, the complete results (including the statistical confounders) of the model adjusted for comorbidity are included in S2 Table. Importantly, it was not possible to adjust the models examining the association between being diagnosed with any psychiatric disorder and the violence outcomes for comorbidity since the employed psychiatric disorder measure simply indicates whether any diagnosis was present. Once again, due to the number of statistical comparisons examined (5 composite psychiatric disorder measures + 1 model adjusted for comorbidity * 8 violence outcomes = 48 comparisons), the employed alpha levels were adjusted using a Bonferroni correction (.05/48 = .00104). Prior to adjusting for comorbidity, all of the composite psychiatric disorder (including the measure of any diagnosis) measures were significant and positively associated with engaging in any violent behavior, indicating that the presence of a given disorder significantly increased the odds of engaging in violent behavior. However, after adjusting for comorbidity, only the presence of a personality disorder (OR = 3.94; 95% CI = 3.03–5.11) or a substance use disorder (OR = 2.23; 95% CI = 1.73–2.87) remained statistically significant.

**Table 5:** Adjusted Odds Ratios for Mental Health Measures and Violent Behavior Measures.

	AOR for Violent Behavior (95% CI)							
	Bullied	Fighting	Domestic Violence	Weapon	Hit Hard	Harass/ Threaten	Injured Someone	Any Violence
**Any Mood Disorder**								
Unadjusted for Comorbidity	**1.68**	**2.53**	**1.78**	1.50	**2.16**	**2.62** ^a^	**1.76**	**1.97** ^a^
	**1.08; 2.63**	**1.42; 4.50**	**1.16; 2.73**	.84; 2.66	**1.36; 3.44**	**1.51; 4.57**	**1.18; 2.61**	**1.46; 2.67**
Adjusted for Comorbidity	1.06	1.54	1.33	.86	1.58	1.69	1.22	1.37
	.66; 1.71	.85; 2.81	.85; 2.08	.45; 1.63	.95; 2.63	.89; 3.19	.79; 1.87	.97; 1.94
**Any Anxiety Disorder**								
Unadjusted for Comorbidity	**1.72**	1.62	**1.59**	**1.96**	1.53	1.70	**1.67**	**1.74** ^a^
	**1.12; 2.62**	.86; 3.06	**1.05; 2.42**	**1.03; 3.74**	.96; 2.45	.96; 3.03	**1.06; 2.62**	**1.26; 2.39**
Adjusted for Comorbidity	1.03	.86	1.15	1.35	1.02	.91	1.13	1.12
	.64; 1.66	.43; 1.69	.74; 1.79	.66; 2.78	.60; 1.73	.50; 1.69	.70; 1.81	.80; 1.59
**Any Personality Disorder**								
Unadjusted for Comorbidity	**5.88** ^a^	**11.20** ^a^	**2.76** ^a^	**4.80** ^a^	**3.34** ^a^	**8.27** ^a^	**4.28** ^a^	**4.31** ^a^
	**4.32; 8.00**	**6.24; 20.11**	**1.85; 4.13**	**2.78; 8.27**	**2.24; 4.97**	**5.13; 13.32**	**2.92; 6.26**	**3.39; 5.48**
Adjusted for Comorbidity	**5.63** ^a^	**10.55** ^a^	**2.50** ^a^	**4.51** ^a^	**2.99** ^a^	**7.65** ^a^	**3.97** ^a^	**3.94** ^a^
	**4.08; 7.76**	**5.60; 19.86**	**1.66; 3.77**	**2.57; 7.90**	**1.95; 4.57**	**4.75; 12.33**	**2.63; 6.00**	**3.03; 5.11**
**Any Substance Use Disorder**								
Unadjusted for Comorbidity	**1.87** ^a^	**2.02**	**1.80**	**2.73** ^a^	**2.29** ^a^	1.44	**2.22** ^a^	**2.35** ^a^
	**1.27; 2.77**	**1.14; 3.58**	**1.13; 2.86**	**1.54; 4.82**	**1.49; 3.52**	.77; 2.69	**1.52; 3.25**	**1.83; 3.01**
Adjusted for Comorbidity	**1.64**	**1.82**	**1.68**	**2.53**	**2.10** ^a^	1.23	**2.09** ^a^	**2.23** ^a^
	**1.01; 2.43**	**1.01; 3.27**	**1.05; 3.77**	**1.44; 4.45**	**1.35; 3.25**	.65; 2.34	**1.41; 3.12**	**1.73; 2.87**
**Any Psychiatric Disorder**								
Unadjusted for Comorbidity	**3.98** ^a^	**11.92** ^a^	**2.34** ^a^	**5.56** ^a^	**3.30** ^a^	**5.97** ^a^	**5.64** ^a^	**3.86** ^a^
	**2.69; 5.87**	**5.04; 28.21**	**1.60; 3.43**	**3.08; 10.03**	**2.24; 4.87**	**3.21; 11.09**	**3.80; 8.36**	**3.04; 4.87**

All odds ratios estimated using sampling weights and were adjusted for the following confounders: mental disability; general health; stressful life experiences; family income; living situation; martial status; sex; race; and age. Models adjusted for comorbidity also include indicators of all other composite psychiatric diagnosis measures. Bolded coefficients have an accompanying 95% CI that does not include 1.00.

^a^ *p* < .00104 (Bonferroni corrected alpha for 48 comparisons)

**Fig 1:**
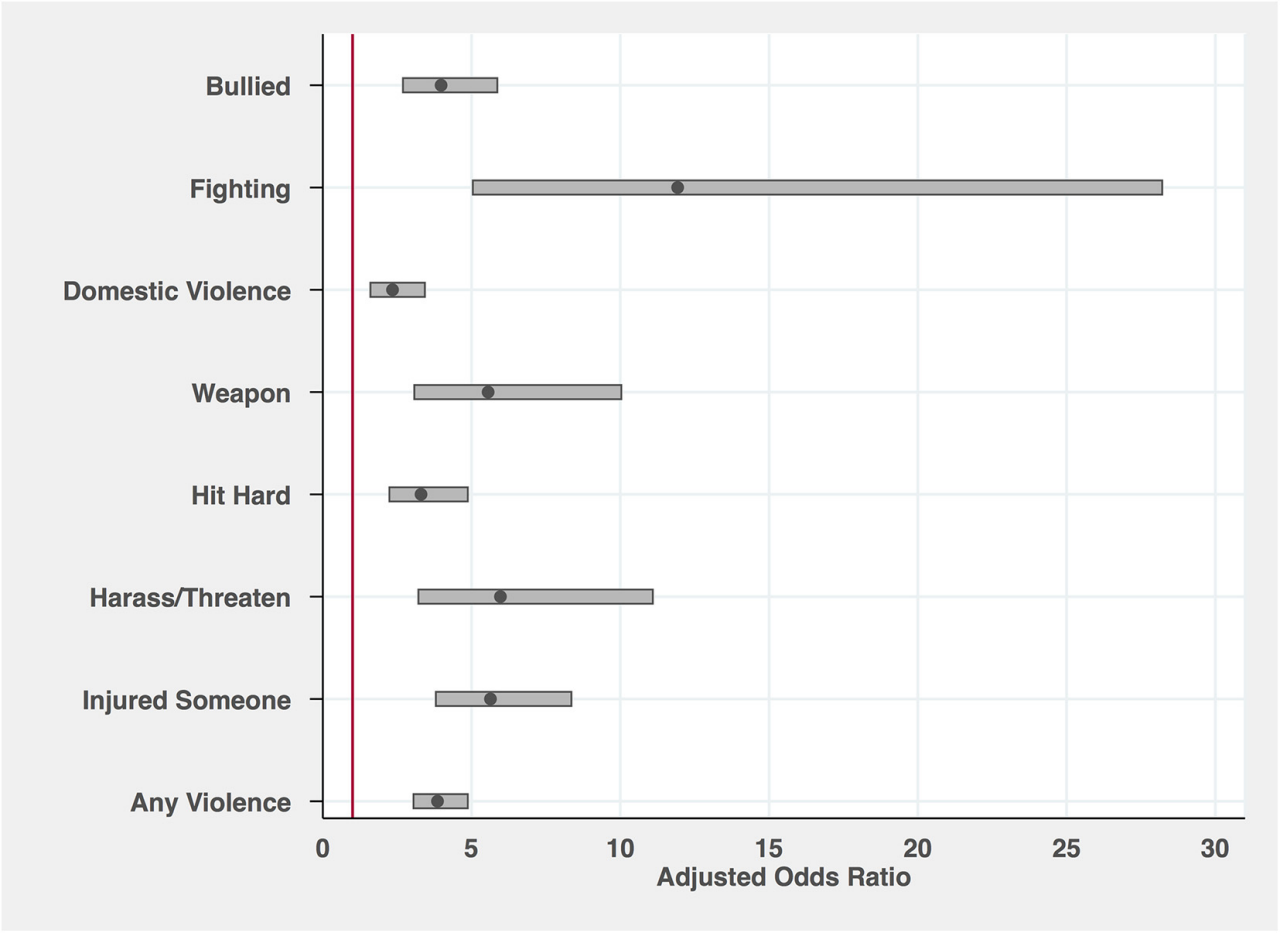
Adjusted Odds Ratios of Violent Behavior for Any Psychiatric Diagnosis. Presents the results of logistic regression models in which each outcome was separately regressed on the composite psychiatric disorder indicator and the following covariates: mental disability; general health; stressful life experiences; family income; living situation marital status; sex; race; and age. The resulting odds ratios are presented as a solid dot and the accompanying 95% confidence intervals are presented in the shaded region surrounding each dot. Standard errors were corrected for sampling procedures using appropriate sample weights. All presented coefficients were significant at the Bonferroni-corrected *p* < .00104 level (48 comparisons).

To visualize the associations between the composite psychiatric disorder measure and the violent behavior outcomes, the resulting odds ratios (adjusted for all included statistical confounders) and 95% confidence intervals are plotted in Fig 1. The solid dots represent the resulting odds ratios and the shaded region displays the surrounding 95% confidence intervals. The results indicated that being diagnosed with any psychiatric disorder significantly increased the odds of bullying someone (OR = 3.98; 95% CI = 2.69–5.87), fighting (OR = 11.92; 95% CI = 5.04–28.21), engaging in domestic violence (OR = 2.34; 95% CI = 1.60–3.43), using a weapon in a fight (OR = 5.56; 95% CI = 3.08–10.03), hitting someone hard enough to need medical attention (OR = 3.30; 95% CI = 2.24–4.87), harassing/threatening someone (OR = 5.97; 95% CI = 3.21–11.09), injuring someone (OR = 5.64; 95% CI = 3.80–8.36), and engaging in any violent behavior (OR = 3.86; 95% CI = 3.04–4.87).

The final step of the analysis compared the associations between the composite psychiatric disorder measures and the composite violent behavior measure across the student and non-student subsamples. The results of the logistic regression models including multiplicative interaction terms between each psychiatric disorder indicator and the college student indicator measure are presented in Table 6. The results revealed significant independent associations between each of the examined psychiatric disorder measures and the violent behavior measure, but none of the examined interaction terms were significant. This pattern of findings provides preliminary evidence suggesting that the associations between psychiatric disorders and violent behavior observed in previous steps of the analysis do not significantly vary between college students and individuals not enrolled in college.

**Table 6:** Models Comparing the Association between Psychiatric Diagnosis and Violent Behavior Measures Across Student Status.

	AOR	95% CI	*p*
**Any Mood Disorder**			
Mood Disorder	**2.37** ^a^	**2.13; 2.64**	**< .001**
College Student	.83	.64; 1.07	.154
Mood Disorder × College Student	.78	.57; 1.06	.109
N	41,400		
**Any Anxiety Disorder**			
Anxiety Disorder	**2.05** ^a^	**1.85; 2.27**	**< .001**
College Student	.80	.61; 1.06	.114
Anxiety Disorder × College Student	.83	.61; 1.13	.238
N	41,400		
**Any Personality Disorder**			
Personality Disorder	**5.26** ^a^	**4.80; 5.77**	**< .001**
College Student	.84	.64; 1.11	.226
Personality Disorder × College Student	.81	.63; 1.06	.120
N	41,400		
**Any Substance Use Disorder**			
Substance Use Disorder	**2.42** ^a^	**2.15; 2.72**	**< .001**
College Student	.86	.66; 1.11	.242
Substance Use Disorder × College Student	.81	.63; 1.04	.103
N	41,400		
**Any Psychiatric Disorder**			
Psychiatric Disorder	**4.00** ^a^	**3.66; 4.38**	**< .001**
College Student	.90	.66; 1.22	.493
Psychiatric Disorder × College Student	.85	.67; 1.08	.178
N	41,400		

All odds ratios estimated using sampling weights and were adjusted for the following covariates: mental disability; general health; stressful life experiences; family income; living situation; martial status; sex; race; and age. Bolded coefficients have an accompanying 95% CI that does not include 1.00.

^a^ *p* < .010 (Bonferroni corrected alpha for 5 comparisons)

## Discussion and Conclusions

With widespread concern regarding mental health and its connection to violence among college students, it is curious that there has not been empirical research examining the link between mental health diagnosis and lifetime violent behavior within a college sample. The current study addressed this gap in the literature by analyzing data drawn from the NESARC. The analyses revealed three key findings. First, when comparing involvement in violent behaviors between college students and those not attending college, college students engaged in significantly lower overall levels of violence for the majority of the examined behaviors, with the only exceptions being harassing/threating someone and physically injuring someone (which did not differ between college students and those in the general population). Despite the overall lower prevalence of violence among college students relative to those not attending college, these findings also revealed that over 21% of college students have engaged in some form of violence previously. These findings align with previous research and seem to indicate that violent behavior is at least somewhat common among college students [30–35].

Second, the analyses also revealed that college students were just as likely to be diagnosed with the vast majority of examined psychiatric disorders as individuals who did not attend college. These findings suggest that nearly 37% of college students have some form of a diagnosable mental health disorder. The overall prevalence of psychiatric disorders among this population indicates that recent calls for improved mental health care on college campuses are warranted [1, 6, 36]. Third, the study examined whether psychiatric disorders were related to violent behavior among college students. The results indicated that seven specific psychiatric disorders were significantly associated with violent behavior within the college student subsample before adjusting for comorbidity and four disorders remained significantly associated with engaging in any violent behavior after adjusting for the comorbidity of multiple disorders. In addition, the presence of any psychiatric disorder was significantly associated with the examined violent behavior measures. Importantly, the detected associations between the psychiatric disorder measures and violent behavior within the student and non-student subsamples were statistically indistinguishable from one another.

Taken together, the results of this study strongly suggest that certain psychiatric disorders are relatively common among college students and that such disorders appear to contribute to a wide range of serious, violent behaviors. The question, of course, is what types of safeguards can be employed to recognize and treat psychiatric disorders among college students. Currently, university campuses do not employ a proactive approach. Rather, the general orientation is that students themselves must seek out counseling and treatment [36, 37], with a large proportion of students failing to properly utilize such services [38–40]. In light of previous research revealing that less than 25% of individuals with psychiatric disorders received any type of treatment [5], this strategy is unlikely to be effective. Universities should begin to consider proactive approaches and screening processes that can be used to help deliver appropriate services to students in need. University-wide campaigns advertising services, providing information sheets, and encouraging students to seek help would be a step in this direction.

The results of previous research have also indicated that more targeted treatment approaches may yield results that are both more efficient and effective [10, 12, 13, 16]. For example, the results of a recent systematic review and meta-analysis of 20 studies examining the potential association between a schizophrenia diagnosis and violent behavior revealed that comorbidity stemming from substance use significantly mediated the association [10]. These findings provide preliminary evidence suggesting that successfully identifying and treating substance use problems may significantly impact this association and ultimately contribute to meaningful reductions in violent behavior among college students. Despite these promising findings, future research should aim to empirically examine the potential role of substance use treatment programs in the reduction of violent behavior among college students.

Although the results of the current study further underscore the need for more resources devoted to the mental health-violence nexus among college students, there are a number of limitations with our study that should be addressed in future research. First, the current study relied on lifetime measures of violent behavior, allowing for the possibility that the examined violent behavior measures preceded the examined psychiatric diagnoses. Future research would benefit from an attempt to better specify causal order. In addition, the examined violent behavior measures were self-reported. While previous research has revealed that self-reported measures of offending are both valid and reliable [41–43], future research would benefit from investigating whether a similar pattern of findings emerges when examining official records of contact with the criminal justice system. More analyses similar to those provided by Pollock et al. [44] will help researchers establish the likelihood of over- or under-reporting violent behavior in a survey setting. As it currently stands, the best evidence suggests respondents are no more likely to under- or over-report their involvement in crime, suggesting self-reports of violent behavior are valid measurement indicators [44].

Third, the current study used a cross-sectional design, making it difficult to establish causality and temporal order (as indicated above). While the NESEARC contains a second wave of data, the current study made use of measures from the first wave of data collection. Due to the amount of time that passed between the collection of the first and second waves of data (approximately 2–4 years depending on the interview date) a large proportion of the college student sample were no longer enrolled in college when the second wave of data was collected. Fourth, the current study identifies associations between the diagnosis of mental health disorders and violent behavior, but due to data limitations, we were not able to identify the specific mechanisms driving this association. Future research would benefit from disentangling the myriad processes that ultimately contribute to individual-level differences in violent behavior stemming from various mental health disorders. Finally, while the analyses performed in the current study were adjusted for a wide range of potential confounding influences, the possibility of additional confounding influences cannot be ruled out. Since the randomization of mental heath and violent behavior is not readily feasible, future research may benefit from estimating models that make use of quantitative matching techniques, such as propensity score matching.

College campuses remain—when compared to other public spaces—relatively safe. Even campuses that are based in some of the most dangerous and violent cities enjoy relatively low crime rates. Despite the overall safety of college campuses, the results of the current study indicate that the overall prevalence of both mental health problems and violent behavior is just as common among college students as their non-student counterparts. These findings further underscore previous calls for additional services to connect college students in need of assistance with effective, evidence-based programming targeting the symptoms of various mental health disorders [1–8]. Such intervention may not only increase the overall quality of life for students with a diagnosable disorder, but may also contribute to the continued safety of college campuses. While the replication of the findings of the current study is still required, providing students with additional resources aimed at identifying and properly treating mental health issues would likely benefit college campuses in a multitude of ways that likely extend beyond safety concerns.

## Supporting Information

S1 Table Unstandardized and Standardized Prevalence for Violence and Psychiatric Diagnosis Measures by College Status. (DOCX)

S2 Table Adjusted Odds Ratios Between Psychiatric Diagnosis Measures and Violent Behavior Measures. (DOCX)
