# Supplementary material for: The Association between Mental Health and Violence among a Nationally Representative Sample of College Students from the United States
Source: PLoS One. 2015 Oct 7;10(10):e0138914. doi: 10.1371/journal.pone.0138914 (PMC4596576; doi:10.1371/journal.pone.0138914)
Supplement: S1 Table — (DOCX) [file pone.0138914.s001.docx]

S1 Table. Unstandardized and Standardized Prevalence for Violence and Psychiatric Diagnosis Measures by College Status

|  | **Not in College** | | | **College Student** | | |
| --- | --- | --- | --- | --- | --- | --- |
|  | **90.07% (N= 39,164)** | | | **9.93% (N = 3,929)** | | |
|  | *Unstandardized* | *Standardized* | *Unstandardized* | | *Standardized* |  |
| **Violence Measures** |  |  |  | |  |  |
| Bullied Others, % | 6.18 | 9.64 | 7.03 | | 8.00 |  |
| Got into Fights, % | 2.86 | 4.55 | 2.47 | | 2.80 |  |
| Domestic Violence, % | 6.78 | 8.23 | 5.38 | | 6.78 |  |
| Used Weapon in Fight, % | 2.71 | 3.63 | 2.55 | | 3.67 |  |
| Hit Someone Injury, % | 5.95 | 10.45 | 6.70 | | 9.75 |  |
| Harass/Threatened Someone, % | 1.59 | 3.20 | 3.08 | | 3.43 |  |
| Physically Injured Someone, % | 4.82 | 8.60 | 7.46 | | 9.14 |  |
| Any Violent Behavior, % | 16.85 | 22.53 | 19.38 | | 21.35 |  |
| **Mood Disorder Diagnosis** |  |  |  | |  |  |
| Major Depression, % | 6.89 | 9.10 | 8.68 | | 8.36 |  |
| Dysthymia, % | 1.86 | 2.03 | 1.58 | | 1.81 |  |
| Manic Disorder, % | 1.61 | 2.54 | 2.12 | | 2.01 |  |
| Hypomanic Disorder, % | 1.04 | 2.56 | 2.25 | | 2.27 |  |
| Any Mood Disorder, % | 8.93 | 11.90 | 11.85 | | 10.95 |  |
| **Anxiety Disorder Diagnosis** |  |  |  | |  |  |
| Panic Disorder, % | 1.53 | 2.04 | 1.67 | | 1.87 |  |
| Panic Disorder w/ Agro., % | 0.56 | 0.75 | 0.59 | | 1.14 |  |
| Social Phobia, % | 2.70 | 3.07 | 3.18 | | 3.09 |  |
| Specific Phobia, % | 7.06 | 8.35 | 7.76 | | 8.19 |  |
| Generalized Anxiety, % | 3.61 | 3.68 | 3.14 | | 4.25 |  |
| Any Anxiety Disorder, % | 12.11 | 13.38 | 12.67 | | 13.32 |  |
| **Personality Disorder Diagnosis** |  |  |  | |  |  |
| Conduct Disorder, % | 1.03 | 1.67 | 1.31 | | 2.11 |  |
| Antisocial Personality, % | 3.52 | 6.28 | 4.73 | | 5.92 |  |
| Avoidant Personality, % | 2.35 | 3.15 | 2.38 | | 2.50 |  |
| Dependent, % | 0.50 | 0.76 | 0.38 | | 0.34 |  |
| Obsessive-Compulsive, % | 7.72 | 8.21 | 9.36 | | 9.93 |  |
| Paranoid Personality, % | 4.38 | 6.31 | 4.75 | | 4.90 |  |
| Schizoid Personality, % | 3.12 | 3.88 | 3.19 | | 3.13 |  |
| Histrionic Personality, % | 1.74 | 3.05 | 2.82 | | 2.71 |  |
| Any Personality Disorder, % | 15.28 | 18.28 | 18.62 | | 19.15 |  |
| **Substance Disorder Diagnosis** |  |  |  | |  |  |
| Alcohol Use, % | 7.76 | 14.50 | 14.97 | | 16.22 |  |
| Drug Use, % | 1.84 | 5.48 | 3.28 | | 3.94 |  |
| Any Substance Use Disorder, % | 8.62 | 16.22 | 16.15 | | 17.34 |  |
| **Any Diagnosis, %** | 30.68 | 35.55 | 38.90 | | 36.77 |  |

Abbreviations: Agro, Agroaphobia

All estimates calculated using survey weights to correct for sampling procedures. Age and sex standardized prevalence presented.
