# Supplementary material for: The Association between Mental Health and Violence among a Nationally Representative Sample of College Students from the United States
Source: PLoS One. 2015 Oct 7;10(10):e0138914. doi: 10.1371/journal.pone.0138914 (PMC4596576; doi:10.1371/journal.pone.0138914)
Supplement: S2 Table — (DOCX) [file pone.0138914.s002.docx]

S2 Table. Adjusted Odds Ratios Between Psychiatric Diagnosis Measures and Violent Behavior Measures

|  | **Bullied** | **Fighting** | **Domestic Violence** | **Weapon** | **Hit Hard** | **Harass/ Threaten** | **Injured Someone** | **Any Violence** |
| --- | --- | --- | --- | --- | --- | --- | --- | --- |
| **Psychiatric Diagnoses** |  |  |  |  |  |  |  |  |
| Any Mood Disorder | 1.06 | 1.54 | 1.33 | .86 | 1.58 | 1.69 | 1.22 | 1.37 |
|  | .66; 1.71 | .85; 2.81 | .85; 2.08 | .45; 1.63 | .95; 2.63 | .89; 3.19 | .79; 1.87 | .97; 1.94 |
| Any Anxiety Disorder | 1.03 | .86 | 1.15 | 1.35 | 1.02 | .91 | 1.13 | 1.12 |
|  | .64; 1.66 | .43; 1.69 | .74; 1.79 | .66; 2.78 | .60; 1.73 | .50; 1.69 | .70; 1.81 | .80; 1.59 |
| Any Personality Disorder | **5.63** | **10.55** | **2.50** | **4.51** | **2.99** | **7.65** | **3.97** | **3.94** |
|  | **4.08; 7.76** | **5.60; 19.86** | **1.66; 3.77** | **2.57; 7.90** | **1.95; 4.57** | **4.75; 12.33** | **2.63; 6.00** | **3.03; 5.11** |
| Any Substance Disorder | **1.64** | **1.82** | **1.68** | **2.53** | **2.10** | 1.23 | **2.09** | **2.23** |
|  | **1.01; 2.43** | **1.01; 3.27** | **1.05; 3.77** | **1.44; 4.45** | **1.35; 3.25** | .65; 2.34 | **1.41; 3.12** | **1.73; 2.87** |
| **Covariates** |  |  |  |  |  |  |  |  |
| Mental Disability | 1.08 | .96 | .92 | .91 | 1.17 | .98 | .98 | 1.03 |
|  | .92; 1.26 | .73; 1.25 | .79; 1.08 | .74; 1.12 | .98; 1.39 | .76; 1.26 | .83; 1.15 | .92; 1.14 |
| General Health | **.80** | .87 | **.73** | .89 | **.73** | **.76** | .86 | **.71** |
|  | **.68; .96** | .67; 1.12 | **.62; .87** | .70; 1.14 | **.60; .89** | **.61; .94** | .73; 1.02 | **.63; .79** |
| Stressful Life Experiences | **1.25** | **1.41** | **1.61** | **1.73** | **1.43** | **1.46** | **1.35** | **1.41** |
|  | **1.09; 1.44** | **1.06; 1.88** | **1.34; 1.93** | **1.34; 2.24** | **1.20; 1.70** | **1.15; 1.85** | **1.14; 1.59** | **1.27; 1.57** |
| Family Income | 1.01 | 1.03 | 1.02 | 1.03 | 1.02 | **1.06** | 1.03 | **1.04** |
|  | .98; 1.05 | .97; 1.09 | .98; 1.05 | .98; 1.08 | .98; 1.06 | **1.00; 1.12** | .99; 1.07 | **1.01; 1.06** |
| Living Situation | .72 | **.39** | .84 | 1.06 | 1.30 | .65 | .65 | .86 |
|  | .48; 1.08 | **.19; .78** | .50; 1.40 | .56; 1.98 | .73; 2.34 | .39; 1.10 | .41; 1.02 | .64; 1.15 |
| Marital Status | **1.48** | 1.50 | .91 | 1.54 | **1.70** | 1.12 | .71 | 1.01 |
|  | **1.00; 2.20** | .75; 2.99 | .57; 1.44 | .82; 2.87 | **1.09; 2.66** | .58; 2.15 | .45; 1.12 | .76; 1.35 |
| Sex | **1.68** | 1.61 | **.59** | **2.85** | **4.48** | 1.60 | **3.24** | **2.28** |
|  | **1.12; 2.53** | .99; 2.62 | **.39; .90** | **1.75; 4.64** | **3.10; 6.48** | .92; 2.79 | **2.31; 4.54** | **1.77; 2.95** |
| African-American | 1.35 | .62 | **1.66** | **4.10** | **2.06** | .95 | 1.18 | **1.60** |
|  | .86; 2.11 | .30; 1.29 | **1.20; 2.30** | **2.38; 7.06** | **1.31; 3.22** | .54; 1.67 | .74; 1.87 | **1.21; 2.12** |
| Native American | **5.06** | 1.33 | 1.94 | **5.37** | **4.06** | 2.29 | .95 | **4.38** |
|  | **1.82; 14.10** | .18; 9.97 | .72; 5.21 | **1.52; 18.94** | **1.66; 9.89** | .40; 13.02 | .23; 4.00 | **1.97; 9.78** |
| Asian | 1.66 | .56 | .36 | .68 | .59 | .75 | .46 | .70 |
|  | .93; 2.97 | .17; 1.87 | .11; 1.18 | .13; 3.48 | .21; 1.62 | .27; 2.07 | .20; 1.07 | .40; 1.25 |
| Hispanic | 1.23 | 1.18 | 1.29 | 1.54 | .94 | .92; 2.79 | .82 | 1.16 |
|  | .77; 1.98 | .58; 2.44 | .84; 1.97 | .71; 3.35 | .56; 1.56 | .50; 1.71 | .48; 1.41 | .84; 1.59 |
| Age | **.85** | 1.07 | 1.20 | 1.30 | .93 | .74 | .97 | .93 |
|  | **.73; .98** | .78; 1.47 | .98; 1.46 | 1.04; 1.63 | .76; 1.13 | .55; .99 | .80; 1.17 | .82; 1.06 |
| N | 3,814 | 3,818 | 3,817 | 3,818 | 3,816 | 3,818 | 3,815 | 3,804 |

All odds ratios estimated using sampling weights and were adjusted for the included covariates. Bolded coefficients have an accompanying 95% CI that does not include 1.00.
